# Substances of abuse and their effect on SAR-CoV-2 pathogenesis

**DOI:** 10.1515/nipt-2023-0004

**Published:** 2023-07-31

**Authors:** Ivy Antwi, Destiny Watkins, Alahn Pedawi, Atheel Ghrayeb, Christine Van de Vuurst, Theodore J. Cory

**Affiliations:** Department of Clinical Pharmacy, College of Pharmacy, University of Tennessee Health Science Center, Memphis, TN, USA

**Keywords:** COVID-19, molecular effect, organ/systemic effect, SARS-CoV-2, substance use disorder

## Abstract

Following the emergence of SARS-CoV-2, various reports suggest that there has been a significant increase in substance abuse due to social distancing and related issues. Several reports have suggested the impact of chronic substance use on individuals’ physiological and psychological health. Therefore, there is a need to know the impact of SARS-CoV-2 on persons with substance use disorders. Individuals with substance use disorders are the most vulnerable groups and are at a high risk of SARS-CoV-2 infection due to their already existing health issues associated with substance use. This review discusses some of the molecular and systemic/organic effects chronic substance use such as alcohol, nicotine, marijuana (cannabis), opioids, methamphetamine, and cocaine have on SARS-CoV-2 infectivity and its potential cause for worsened disease outcomes in persons with substance use disorder. This will provide healthcare providers, public health policies, and researchers with the needed knowledge to address some of the many challenges faced during the Covid-19 pandemic to facilitate treatment strategies for persons with substance use disorders.

## Introduction

SARS-CoV-2 is a highly pathogenic member of the coronavirus family [1]. Belonging to the same family, SARS-CoV, and MERS have similar symptoms as SARS-CoV-2 including cough, chest pain, shortness of breath fever, and pneumonia, were not classified as a pandemic due to their lower mortality rate [1]. The World Health Organization (WHO) officially declared SARS-CoV-2 a global pandemic on March 11, 2020, after reporting over 118,000 cases in 114 countries with 4,291 deaths [2].

**Table 1:** Summary: substances of abuse and their effect on SAR-CoV-2 pathogenesis.

Drugs of abuse	Summary
Alcohol	COVID-19 outcomes are worsened in people with alcohol use disorders due to immune system dysfunction, increased risk of aspiration pneumonia, thrombosis, cardiovascular disease, and liver metabolic diseases associated with alcohol use
	Heavy alcohol use significantly decreases lung macrophage, lymphocyte cytokine production and decreases neutrophil recruitment, all of which alter the host’s immune response to pathogens
Nicotine	Increased ACE2 expression in tobacco smokers may result in more cellular entry points for SARS-CoV-2
	Cigarette tar and tobacco smoke decrease lung function and capacity over time by damaging the lining and alveolar sacs of the lungs increasing the risk of respiratory distress from COVID-19
	Tobacco functions as an immunomodulator that can significantly suppress the immune system by reducing antibody responses and T-cell proliferation, thereby increasing the susceptibility of tobacco users to acute viral infections
Marijuana	Marijuana use increases the likelihood of cough, sputum production, wheezing, chest sounds, chronic bronchitis, and cardiovascular complications
	Marijuana can modulate the immune system by acting on T-cells, B cells, macrophages, and lymphocyte receptors, decreasing immunity and increasing susceptibility to viral infections such as COVID-19
Opioids	COVID-19 and opioid exposure may have similar disease outcomes including respiratory depression and hypoxia, and the combination of these two could lead to an aggravated complication in neuro-immunity and respiratory depression
	Acute kidney injury from opioid use is commonly accompanied by respiratory failure, which is associated with poor COVID outcomes including ventilation, renal replacement therapy, and death
Methamphetamine	Free radical formation from methamphetamine use causes pulmonary toxicity which impairs lung function
	Cognitive impairments caused by a loss of dopamine and serotonin from chronic methamphetamine use could contribute to the mood and sleep disorders seen in those suffering from long COVID
Cocaine	Patients with COVID-19 and cocaine use disorders experience an increase of pro-inflammatory and decreased anti-inflammatory cytokines
	Cocaine use affects many organs throughout the body, including the cardiovascular, CNS, and respiratory systems, causing a higher risk of susceptibility
	The synergistic effect of infection from SAR-COV-2 and the consumption of cocaine is prothrombic, resulting in a hypercoagulable state, and increasing the risk of myocardial infarction
Cannabinoids	Cannabidolic acid and cannabigerolic acid, may block infection of human cells by SARS-CoV-2 expressing the spike protein
	CBD can reduce proinflammatory cytokines IL-2, IL-6, IL-1α and β, interferon gamma, and inducible protein-10 that have been associated with SARS-CoV-2

SARS-CoV-2 can infect all immune cells (monocyte, dendritic cells, macrophages, lymphocytes) leading to hypercytokinemia (cytokine storm) resulting from immune dysregulation [3–7]. The excessive cytokine release in the presence of SARS-CoV-2 in other parts of the body especially the lungs, leads to acute respiratory distress syndrome [8]. Not only does SARS-CoV-2 affect the lungs, but researchers and clinicians have reported implications in some major organs and systems including the central nervous system, heart, liver, kidney, gastrointestinal tract, and blood vessels [9–11].

The COVID-19 pandemic has not only impacted the health of people but health care systems and policies around the world [12, 13]. These impacts range from social distancing, lockdowns, financial constraints, etc., in some way, impacted the physiological and mental well-being of people [13, 14]. Approximately 45 % of US adults have expressed stress and worry over the virus, which has impacted their mental health negatively [15]. These factors related to the pandemic considerably impacted the consumption of substances, especially in people with substance use disorders which may affect their health negatively [16].

A recent report by the Center for Disease Control and Prevention (CDC) suggests that the number of deathsfrom substance overdose in the United State is over 932,000 since 1999 [17]. The most common substances abused in the US include alcohol, opioids (prescription or illicit), marijuana, tobacco (nicotine), cocaine, and methamphetamine [18]. The increase in distress and anxiety associated with the COVID-19 pandemic brought about a general increase in cannabis consumption (6–8 % in adults), alcohol consumption (10–18 %), and other substances (3 %) [19]. Data suggest that people with prior substance use disorders (SUD) increased their consumption during the pandemic while others consumed substances in order to cope with the social impacts the pandemic brought [19].

People with substance use disorders are often subjected to chronic illnesses such as COPD, arrhythmias, myocardial infarction, hypertension, diabetes, and cardiac insufficiency [20–22], which are all risk factors associated with COVID-19 infections. It is probable that individuals with substance use disorders have worsened disease outcomes [23–25].

Currently, there is little information regarding the direct effect SARS-CoV-2 has on individuals with substance use disorder in terms of the individual’s organs and systems. Therefore, in this review, we discuss the molecular and systemic/organic effects chronic substance use such as alcohol, nicotine, marijuana (cannabis), opioids, methamphetamine, and cocaine have on SARS-CoV-2 infectivity, and how SARS-CoV-2 may worsen disease outcomes in persons with substance use disorder based on the limited published articles Table 1.

## Effects of alcohol use on SARS-CoV-2

The surge of the COVID-19 pandemic resulted in disruption to health care and an increase in alcohol use, resulting in serious health repercussions in people with alcohol use disorders (AUD) [26, 27]. Worsened disease outcomes were observed, especially in people with alcohol-associated liver disease [16]. This section of the paper discusses what happens at the molecular and organic/systemic levels when people with alcohol use disorders are infected with SARS-CoV-2.

### Molecular effects

Excessive alcohol consumption can cause immune system impairment, liver cirrhosis, and other complications that can be associated with worsened outcomes in respiratory disorders [28–30]. As a result, severe and worsened progression of COVID-19 in individuals with alcohol use disorders was hypothesized by Dubey et al. and Muhammad et al. [31, 32]. Dubey et al. suggested that there may be a synergistic worsening of COVID-19 outcomes in people with alcohol use disorders due to immune system dysfunction, increased risk of aspiration pneumonia, thrombosis, cardiovascular disease, and liver metabolic diseases associated with alcohol use [31]. Muhammed et al. reported upregulation of proinflammatory markers CCR2, DPP9, HSPAIL, TYK2, OAS1, ACE2, and TMPRSS2 in brain tissues of alcohol use disorder patients who died while hospitalized with severe COVID-19 outcomes as compared to non-alcohol using patients [32]. In this report, they observed an upregulation of HSPAIL, a gene involved in host epigenetic regulation that leads to increased SARS-CoV-2 replication and the OAS1 gene associated with viral infection susceptibility. OAS1 and HSPAIL are genes associated with increased receptiveness to viral infections while CCR2 and TYK2 are also important drivers of inflammation. Based on these observations, they hypothesized that individuals with alcohol use disorders are at increased risk of developing severe neurological complications when exposed to SARS-CoV-2 compared to those without alcohol use disorder [32, 33]. Friske et al. reported upregulation of ACE2 and TMPRSS2, the entry points of SARS-CoV-2, and the anti-inflammatory gene MAS, which is activated upon infection in the lungs of chronic alcohol-exposed rat models [34]. They also reported upregulation of ACE2 in other organs including the liver, heart, kidney, ileum, and brain in the alcohol-exposed group. Although upregulation of the entry point for SARS-CoV-2 may lead to increased infectivity and reduced immunity to viral infectivity in these organs, the authors did not assess the potential for worsened outcomes [34–37]. Huang et al. reported a Network Meta-Analysis suggesting that ethanol exposure may increase SARS-CoV-2-induced systemic inflammation by altering important inflammation mediators [38]. This report suggests that ethanol increases the effect of SARS-CoV-2 infection on cellular metabolism, the hepatic fibrosis signaling pathway, inflammation, cellular homeostasis, and neuroinflammation. This was observed following increased activity of cytokines (IL-6, TNF, IL-1b), transcription factors including STAT and JUN, and the inhibition of anti-inflammatory mediators’ activity such as glucocorticoid receptors [38].

### Systemic/organ effects

As previously stated, long-term use of alcohol can be extremely detrimental to an individual’s health [28]. Alcohol use is one of the leading causes of acquired immunity disorders and can be linked to being a major risk factor for SARS-COV-2 contraction and severity [39, 40]. Healthy lungs possess protective mechanisms such as alveolar macrophages, which trigger an immune response and mediate the influx of neutrophils into the lungs [30]. This process plays a pivotal role in the clearance of viral pathogens. Heavy alcohol use significantly decreases lung macrophage and lymphocyte cytokine production and decreases neutrophil recruitment, all of which alter the host immune response [30]. This causes damage to the alveolar barrier, altering the integrity and making it more permeable to viruses [41]. Phagocytosis of viruses is also impaired in heavy alcohol users by depleting glutathione stores in the lungs [28]. This results in increased susceptibility to oxidative stress due to impaired detoxification and clearance of toxic oxidants, increasing the risk of acute respiratory distress syndrome (ARDS) [28]. Furthermore, alcohol alters the flora in the oropharynx, facilitating the colonization of Gram-negative bacteria. Alcohol causes cough and gag reflex suppression, which increases the risk of bacteria being aspirated. Collectively, these mechanisms contribute to respiratory system decline, increasing the risk for SARS-CoV-2 [30, 42]. SARS-CoV-2 produces a strong inflammatory response, often leading to hypercytokinemia [28]. This hyperactive inflammatory response is one of the leading causes of systemic organ failure in SARS-CoV-2 [42]. Heavy alcohol users have higher baseline concentrations of pro-inflammatory markers and lower levels of essential micronutrients. This predisposes them to poorer prognoses and more severe exacerbations of inflammatory diseases, like SARS-COV-2 [40, 43].

Although these findings suggest a possible worsened outcome in persons with AUD it does not necessarily support a direct worsened disease outcome in these individuals. Research and experimental data must be published to indicate the direct association between SARS-CoV-2 and alcohol use disorder causing worsened disease severity.

## Effect of nicotine use on SARS-CoV-2

Nicotine, while one of the leading causes of preventable death in the United States, also saw an increasing trend of use during the pandemic [44]. The addictive use of nicotine is associated with increased morbidity, often leading to cancer and death. Moreover, SARS-CoV-2 can synergistically deteriorate lung functions further when nicotine is abused in persons with nicotine use disorders [45]. Due to this reason, it is necessary to examine the effect nicotine use disorders have on SARS-CoV-2 at both the molecular and organic levels.

### Molecular effects

Since the emergence of SARS-CoV-2 in 2019 many diverse factors have been linked to the severity of the disease among tobacco smokers since they both impact the respiratory system [45–47]. Over the years, studies on the effect of tobacco smoking have established the fact that it compromises the immune and respiratory systems [48, 49]. However, the effect of tobacco smoking on SARS-CoV-2 infection and its impact on COVID-19 remains surprisingly controversial [50]. The major entry receptor of SARS-CoV-2 to host cells is via the human angiotensin converting enzyme-2 [4, 51, 52]. Overexpression of ACE2 in lower respiratory tract epithelial cells has been observed in current tobacco smokers due to nicotine induced ACE2 expression [52–55]. Increased ACE2 expression in tobacco smokers may result in more cellular entry points for SARS-CoV-2 [55]. Maggi et al. reported a direct link between nicotine exposure to worsened outcomes in SARS-CoV-2 infection [56]. In this report, they observed a rapid and long-lasting increase in protein and gene expression of the ACE2 receptor when they exposed the human pulmonary adenocarcinoma epithelial cell line A549, which naturally expresses low levels of ACE2, to nicotine [56]. Specifically, they noticed that exposure to nicotine in this cell line not only upregulated ACE2 expression [54, 57] but also upregulated α7-nAChR expression via the activation of MAPK/ERK, induced cell proliferation, and cell migration [58, 59]. In terms of its effect on SARS-CoV-2, it increases pathogen replication and transcription of viral proteins [59], thereby increasing the cytopathic effect of SARS-CoV-2. These findings suggest that nicotine increases SARS-CoV-2 infectivity [56, 60]. The idea that nicotine may present a cause for worsened SARS-CoV-2 infection was investigated in another study by looking at the mechanism involved in increased infectivity [57]. In a work presented by Lupacchini et al., they observed that through the induction of the α7-nAChR under conditions that mimic human smokers, nicotine increased cell viability, upregulated phospho-p38, and overexpressed Ki67, EGFR, and pEGFR [58]. They also observed a reduction in ATP [61], p-53, and phospho p53 levels [62], increases in Ca2+ basal concentration [63], and an increase in VEGF. All these findings suggest the involvement of nicotine in the upregulation and severity of SARS-CoV-2 infection [58].

However, some reports suggest that nicotine may reduce inflammation via its interaction with the nAChR, and nicotine itself may prevent the attachment of SARS-CoV-2 to ACE2, therefore protecting host cells against SARS-CoV-2 infection [64–66]. Farsalinos et al. performed a study based on molecular modeling and docking (*in silico*) where they identified an interaction between nAChRs and a toxin-like sequence in the receptor-binding domain of SARS-CoV-2 spike glycoprotein [66]. They observed that the interaction between the glycoprotein of SARS-CoV-2 and nAChRs may lead to a cytokine storm and a hyper-immune response resulting from crosstalk between the nicotine cholinergic system (NCS) and the cholinergic anti-inflammatory pathway. Based on clinical findings that suggest a low rate of hospitalization among tobacco smokers in COVID-19 hospitalized patients, but higher rates of adverse effects when hospitalized, they hypothesized that nicotine and other cholinergic agonists can restore the NCS dysregulation and the function of the cholinergic anti-inflammatory pathway [66, 67]. Although this study shows that nicotine may help restore NCS dysregulation caused by SARS-CoV-2, this study was solely theoretical with no *in vivo* or *in vitro* experiment to confirm the interaction between nAChRs and SARS-CoV-2 [65, 67, 68].

### Systemic/organ effects

Nicotine has well-known adverse effects on organs throughout the body, particularly the lungs, which can impact respiratory and immune function [69]. Damage to these organs can result in an increased risk of SARS-CoV-2 [70]. Studies have focused attention on the ACE2 receptor, and while some studies have suggested that tobacco may be protective against SARS-CoV-2, other studies have shown different results [69, 71]. Previous studies suggested that nicotine upregulates the ACE/angiotensin II (ANG II)/ANG II type 1 receptor axis while downregulating ACE2, resulting in SARS-COV-2 protection [71, 72]. However, more recent studies have shown that tobacco smokers have an increased expression of ACE2 in bronchial epithelial cells in the respiratory tract, which may promote SARS-COV-2 entry and proliferation through co-expression with nicotinic receptors [69, 70]. Cigarette tar, a byproduct of tobacco combustion, can also induce lung damage by damaging cilia in the lungs [70]. This reduces the surface area between capillaries, decreasing the capacity of the arteries to transport oxygenated blood. With low blood oxygen levels being a strong predictor of poor COVID-19 outcomes, this closely ties cigarette use with more severe COVID-19 symptoms [73]. Tobacco smoke is known to activate and modify immune activity in the lungs, which can lead to the development of inflammatory lung diseases, including, COPD, asthma, and other lung diseases, putting tobacco users at a higher risk for contracting infections [71]. Lung function abnormalities demonstrated by smokers when compared with lifelong nonsmokers include reduced forced expiratory volume in 1 s (FEV1), peak expiratory flow (PEF), and the ratio of FEV1 to forced vital capacity (FVC, FEV1/FVC) [74]. Individuals with COPD also have deficiencies in these lung function tests, specifically spirometry [75]. COPD is the single greatest predictor of comorbidity for severe COVID-19 leading to ICU admission, supporting the idea that smoking increases the risk for viral infections, such as SARS-COV-2 [75]. Additionally, tobacco functions as an immunomodulator that can significantly suppress the immune system by reducing antibody responses and T-cell proliferation, thereby increasing the susceptibility of tobacco users to acute viral infections [70]. Immune suppression due to nicotine, particularly through the decrease in CD8+ T-cells that facilitate rapid resolution of acute viral infections, increases the susceptibility of smokers to viral infections [76]. Multiple components of tobacco decrease immune function, and individuals who use tobacco are at an increased risk of susceptibility to infection with SARS-CoV-2.

The findings observed through published articles and data suggest detrimental and potentially beneficial effects of nicotine use in COVID-19. However, this confirms the need for more substantial evidence to support a worsened disease outcome of SARS-CoV-2 in people with nicotine use disorders.

## Effect of marijuana (cannabis) use on SARS-CoV-2

In 2018, an estimated population of 192 million people used cannabis worldwide [77]. This makes cannabis the third most-used substance following alcohol and tobacco [77]. Prolonged usage of marijuana which can be associated with impaired respiratory function, cardiovascular diseases, and adverse psychological and mental health issues must be reviewed when discussing the impact of COVID-19 on persons with cannabis use disorder (CUD) since SARS-CoV-2 also impacts these systems [78].

### Molecular effects

Recent data suggests increased use of cannabis during the COVID-19 lockdown period [79]. Unlike alcohol and nicotine abuse, where experimental models have been utilized to see the impact of the drug on the SARS-CoV-2 infection at the molecular level, no *in vitro* experiments have been done on marijuana use at the time of this review. However, there have been reports suggesting the possible increased susceptibility to SARS-CoV-2 in individuals with cannabis use disorders based on the cellular or molecular complications associated with cannabis use [76, 80], [81], [82]. Marijuana has previously been found to be an immunosuppressant that suppresses the activation of proinflammatory cytokines such as IL-6, IL-1α, TNFα, and IL-1β, as well as T lymphocytes, which are essential mediators in the immune response to pathogens [80]. Due to the downregulation of these key mediators, host cells may be more susceptible to viral pathogens such as SARS-CoV-2 [76]. As an immunomodulator, marijuana works by modulating immune function and the brain [76, 80]. Chronic obstructive pulmonary disease, is a major risk factor for complications associated with worsened COVID-19 outcomes in tobacco smokers [81], has also been suggested to have an evidential risk factor linked to marijuana smokers [82].

Other studies of cannabis products on SARS-CoV-2 suggest a potential therapeutic effect [83–88]. The downregulation of COVID-19 associated proinflammatory cytokines (cytokine storm) has been reported as an aspect to explore in the treatment of the disease [89–92]. Many reports have suggested a decrease in proinflammatory cytokines and chemokines, downregulation of inflammatory pathways associated genes, inhibition of SARS-CoV-2 targeted genes, and inhibition of viral replication when cannabis or its related products were experimentally used in *in vitro* analysis [86, 90, 93], [94], [95], [96]. IL-6 and TNFα, key mediators in a cytokine storm, were reported to be downregulated in a WI-38 lung fibroblast cell line model [97]. They also saw downregulation of COX2, CCL2, and other cytokines involved in inflammatory pathways [97]. Similarly, Anil et al. reported a reduction of IL-6 and IL-8, prominent cytokines involved in cytokine storms in severe COVID-19 patients [98]. They reported an altered inflammatory cytokine response [99] after a 4-h exposure of 5 μg/mL cannabis extract to an alveolar epithelial (A549) cell line, suggesting an anti-inflammatory response to cannabis [98, 100, 101]. In addition to the response observed for IL-6 and IL-8, inflammatory chemokines CCL2 and CCL7 which are found in the bronchoalveolar fluid of severe COVID-19 patients [102] were also observed to downregulate after 6 h treatment of alveolar epithelial with cannabis [98] and ACE2 expression was also found to be downregulated after 4–6 h post cannabis treatment [98, 103]. Treatment with Cannabis Sativa extract also increased the expression of CD36 and FcγRII type II receptor, which are involved in the initiation of macrophage phagocytosis [104, 105], suggesting that cannabis may aid in the clearance of respiratory viruses including SARS-CoV-2 at the initial phase of infection [98]. The downregulation of ACE2 expression and the serine protease TMPRSS2, which serves as a critical entry point for SARS-CoV-2 were seen in a report by Wang et al. [94]. Cannabidiol has also been shown to inhibit SARS-CoV-2 viral gene expression and replication *in vitro* in lung epithelial cells and upregulate antiviral signaling pathway induced by interferon expression [84, 85, 87].

Although some cannabis products may have potential therapeutic properties against SARS-CoV-2, there are side effects that need to be regarded such as a risk of drug interactions with other COVID-19 drugs, and the route of drug administration like vaping which can be detrimental to health [90].

### Systemic/organ effects

During the SARS-CoV-2 pandemic, there has been a surge in marijuana use [106]. While medicinal marijuana may have positive health effects on COVID-19, other studies suggest that marijuana use may result in a higher risk of SARS-CoV-2 acquisition with more severe symptoms [107]. Specifically, chronic marijuana use is associated with immunosuppression, cardiovascular deficiencies, and respiratory tract dysfunction, all of which increase the risk of SARS-CoV-2 [108, 109]. Although individuals who use marijuana have a lower odds ratio of contracting SARS-CoV-2 than opioids, tobacco, and alcohol, the risk is five times greater than in people who do not use substances of abuse [107]. Studies have shown that marijuana and other psychoactive substances modulate the immune system by acting on T-cell, B-cell, macrophage, and lymphocyte receptors, which decrease immunity and increase susceptibility to viral infections [110]. Marijuana has been shown to alter the hypothalamic-pituitary-adrenal (HPA) axis, which plays a significant role in orchestrating immune responses. HPA axis impairment decreases the regulation of pathogens [111]. Additionally, marijuana inhibits T-cell activation by increasing immunosuppressive cytokine production and decreasing the expression of T-cell activating cytokines, which decreases the clearance of pathogens. Thus, marijuana dependent immunosuppression of macrophages, T-cells, and B-cells puts individuals at greater risk of contracting SARS-CoV-2 [109]. With the increasing use of cannabis throughout the population, there are more studies associating cannabis use with cardiovascular complications including acute coronary syndrome, lethal cardiac arrhythmias, and ischemic stroke [107]. Because preexisting cardiovascular disease is a strong predictor of poor disease outcomes in SARS-CoV-2, marijuana use may also be associated with a higher SARS-CoV-2 risk [112]. Furthermore, cannabis use has been reported to increase the risk of ischemic stroke, particularly in healthy young patients. This is a reason for concern given increased use of cannabis in adolescents [113]. Because marijuana users have an increased risk of developing arterial thromboses at baseline, cannabis use may result in worsened outcomes in SARS-CoV-2 despite optimal treatment [114]. Marijuana is used most commonly via inhalation, resulting in similar effects on the lungs as tobacco, including increased cough, sputum production, hyper-inflammation, and upper lobe emphysematous changes [108]. Although it does not seem to contribute to the development of chronic obstructive pulmonary dysfunction (COPD), there is mixed data about the effects that smoking marijuana has on pulmonary function [108]. Data shows that respiratory symptoms are uniformly increased in marijuana smokers. In the NHANES III study, both marijuana and tobacco smokers had an increased likelihood of cough, sputum production, wheezing, chest sounds, and chronic bronchitis [115]. This indicates that smoking marijuana compromises the respiratory system. Therefore, it is likely that since SARS-CoV-2 primarily targets the respiratory system, marijuana smoke will increase SARS-CoV-2 risk.

In terms of the beneficial and detrimental effects, marijuana and nicotine behave similarly as both are inhaled substances. Nonetheless cannabis use disorder could potentially pose a risk factor in COVID-19 patients since long term use of marijuana has increased the morbidity rate. Evidently, more experimental research needs to be conducted to associate marijuana use with the potential for worsened disease outcomes in these individuals.

## Effect of opioid use on SARS-CoV-2

A global study conducted in 2016 indicates that about 26.8 million people live with opioid use disorders (OUD) of which more than 100,000 die annually including over 47,000 people in the USA [116]. Similar to other substances of abuse, dependence on opioids can be detrimental to an individual’s health. Therefore, a study of the molecular and organ impacts SARS-CoV-2 can have on these vulnerable individuals needs to be discussed.

### Molecular effects

Research has shown an increased risk of severe COVID-19 associated with opioid use [117–119]. Individuals with opioid use disorders are already vulnerable to poor health and the emergence of SARS-CoV-2 has further put these individuals in a compromised situation [120].

Although there have not been specific studies conducted on the molecular effects of opioid abuse on SARS-CoV-2 infection, many published articles have established a correlation between the effect of opioid abuse on individuals’ health and the risk that may be presented when people with opioid use disorders (OUDs) are infected with the virus [118, 121, 122]. A report published by Willner et al., suggested that COVID-19 and opioid exposure may have similar disease outcomes including respiratory depression and hypoxia, and that the combination of these two could lead to an aggravated complication in both neuro-immunity and respiratory depression [123, 124]. Opioids stimulate increased levels of pro-inflammatory and neurotoxic cytokines such as IL-6, IL-1β, and TNFα as well as anti-inflammatory and neuroprotective cytokines like IL-10, TGF-β, and BDNF when they bind to opioid receptors in the central nervous system [125, 126]. Similarly, stimulation of inflammatory damages has been observed in some COVID-19 patients diagnosed with neurological symptoms, including encephalitis that may be due to neuroimmunological responses [123, 127]. Due to these similar inflammatory damages to the central nervous system, a combination of opioid abuse and COVID-19 may lead to detrimental health risks [127].

Another study suggests that opioid users are at an increase from COVID-19 due to the suppression of immune functions by opioids [119]. Opioids inhibit leukocyte recruitment, cytokine secretion, and have a destructive effect on innate and adaptive cells. A study by Zhao et al. in SARS-related infected mice showed that inefficient T-cell activation may potentiate lung injury [128]. This relationship between opioid users and SARS-CoV-2 may worsen immunological disease outcomes in persons with OUD.

### Systemic/organ effects

Opioids can lead to both respiratory depression and kidney damage. COVID-19 can also cause severe acute respiratory infections in many patients [129]. There is also a high prevalence of kidney impairment in hospitalized COVID-19 patients [130]. Taken together, there is an increased risk for poor prognosis and death in patients with COVID-19 who abuse opioids [129, 130]. Opioid-related respiratory depression may result in hypoxemia [119]. As early as 6 months after starting opioid use, the risks of respiratory depression can range from 1.9 to 83.4 % [131]. COVID-19 additionally can cause hypoxemia secondary to viral pneumonia, and one large study showed the frequency of hypoxemia in hospitalized patients was 20.4 % [24]. This demonstrates the potential for increased risks of opioid-related respiratory depression with concomitant hypoxemia from viral pneumonia. Opioid use in patients who become infected with COVID-19 may result in an increased risk of adverse respiratory outcomes [119]. The usage of opioids leads to multiple complex interactions throughout various body systems, particularly the endocrine and nervous systems, altering both the sympathetic and parasympathetic autonomic nervous system, so the effects of opioids on renal function may increase the risk of hospital death [130]. Heroin use is linked with a higher risk of CKD progression [132]. This is important for patients hospitalized with COVID-19 and who are also receiving nephrotoxic agents in the hospital, because it may worsen their prognosis. Therefore, it is important to recognize patients with opioid use early in order to improve their chances of recovery [133]. In addition to chronic kidney disease (CKD) causing poorer prognoses, acute kidney injury (AKI) is also a major concern for increasing the risk for SARS-CoV-2 complications in patients with opioid use disorders. AKI with opioid use develops through a mechanism of multi-organ failure from respiratory depression, hypoxia, and volume depletion with or without rhabdomyolysis [134]. Also, an overdose of opioids can result in AKI as a consequence of different mechanisms such as dehydration, hypotension, rhabdomyolysis, and urinary retention [135]. The significance of AKI is described in a study that showed the relationship between AKI and COVID-19 and found that AKI occurs early and is accompanied by respiratory failure, which is associated with poor COVID outcomes including ventilation, renal replacement therapy, and death [89]. Therefore, kidney damage caused by opioid use can further increase the user’s risk for AKI with COVID-19 infection and lead to poor recovery outcomes for these patients. This supports that respiratory depression and kidney damage caused by opioids significantly increase the risks of a poor prognosis and death with SARS-CoV-2 infection.

The published data suggest a negative correlation between OUD and COVID-19, although there is relatively little data to date there is the need for distinct evidence to support the potential cause for worsen disease outcome in persons with OUD as these populations are vulnerable and have a high risk of death.

## Effect of methamphetamine use on SARS-CoV-2

Methamphetamine is a common drug of abuse used by people regardless of age. Methamphetamine use is usually preceded by a previous drug abuse history with opioids, alcohol, cocaine, or heroin. A study conducted from December 2019 to November 2020 during the early stages of the COVID-19 pandemic found that methamphetamine use increased 37 % from the previous year [136]. People living with substance use disorders have been shown to be significantly more vulnerable to complications arising from COVID-19 infection due to respiratory and cardiovascular from substance abuse especially due to methamphetamine [137]. Methamphetamine, which has several forms such as powder or an oily substance, is most commonly smoked and in chronic users has been shown to cause lung complications such as pulmonary toxicity [137] and neurotoxicity [138].

### Molecular effects

Methamphetamines euphoric effects are caused by monoamines released during use that act on the dopaminergic, noradrenergic, and serotonergic pathways in the neurological system. Dopamine then can target central pathways such as the nigrostriatal pathway and the mesocortical and mesolimbic circuit [139]. Distribution of these monoamines is not only limited to the neurological system but others which enhances methamphetamines systemic effect.

Methamphetamine can impact the immune system in a few ways. Altering the efficiency of B cells and disrupting communication between the innate and adaptive cells by cytokines may delay response to pathogens such as COVID-19. Methamphetamine can reduce the numbers of circulating immune cells such as dendritic cells, monocytes, natural killer cells and macrophages, predisposing these users to a higher risk of infection for all viruses, including SARS-CoV-2 [140].

Methamphetamine causes damage to the lungs by increasing oxidative stress [138]. In conditions of oxidative stress, dopamine (DA) can oxidize to form superoxide and hydrogen peroxide, which can then form the hydroxyl radicals [138]. These radicals produced are implicated in methamphetamine induced pulmonary toxicity. DA oxidation forms DA quinones which participates in addition reactions with sulfhydryl groups on free cysteine. This reaction can cause a cascade of reactions including decreasing antioxidant concentrations and can result in decreased lung function over time [138]. Lung function is critically important to maintain in patients with COVID-19 due to the increased risk of mortality with impaired lung function this becomes especially important in those with a previous history of lung damage such as those with chronic methamphetamine use. COVID-19 also causes respiratory stress through similar mechanisms from methamphetamine. Monocytes and macrophages play a critical role in the inflammatory reactions with severe COVID-19 infection [141]. These immune cells release large amounts of pro-inflammatory cytokines such as IL-6 and IL-8 [141]. Increases in the same pro-inflammatory cytokines released in COVID-19 and methamphetamine use could predispose methamphetamine users to an increased risk of developing respiratory distress from COVID-19.

Methamphetamine causes an increase in inflammatory markers such as LC3 [137]. Further, it causes infiltration of inflammatory cells into the lungs causing thickening of alveolar sputum, compacting lung parenchyma, and reduction in the amount of alveolar sacs [137]. An increase in autophagic regulator protein LC3 causes cellular apoptosis by decreasing anti-apoptosis mediated BCL2. Cellular apoptosis in alveolar epithelial cells has been shown to cause pulmonary toxicity in chronic methamphetamine users [137]. SARS-CoV-2 damages the alveolar-capillary barrier by loss of surfactant protein expression causing injury to alveolar epithelial cells, endothelial cells, respiratory epithelial basal cells, and damages to tissue repair processes [142].

Neurodegenerative changes in methamphetamine users are also commonly seen in those with chronic use. Changes such as a loss of dopamine transporters, serotonin transporters, and a loss of dopamine levels have been found in brains of methamphetamine users [143]. Brain scans of this population also show a change in the brain’s anatomy regarding amounts of white and gray matter [143]. The SARS-CoV-2 virus has also been found to impact neurotransmitters such as dopamine and serotonin. SARS‐CoV2‐induced downregulation of ACE2 expression is accompanied by changes in both reduced dopamine and serotonin production, which is exacerbated by chronic use of methamphetamine. Loss of dopamine and serotonin in both disease states could increase the risk of neurodegenerative changes seen in patients with COVID-19 [14].

Neurotoxicity induced by methamphetamine is caused by a decrease in DA, DA transporter, serotonin vesicular monoamine transporter type 2 (VMAT2), and tyrosine hydroxylase [143]. Due to a decrease in VMAT2, an increased risk of neurological conditions such as Parkinson’s are found to be more common in methamphetamine users versus non-methamphetamine users [138]. The use of alcohol in methamphetamine users may further exacerbate neurotoxicity associated with methamphetamine. One study found that dopamine levels decreased by 90 % when alcohol use was recently prior to the use of methamphetamine [144]. Alcohol can induce COX2 in the brain and COX2 has been linked to cause neurotoxicity with methamphetamine use [144].

### Systemic/organ effects

Methamphetamine is an amphetamine analog most commonly smoked using various non-sterile methods with some exposing users to dangerous unknown drugs and bacteria. Chronic use has been associated with immunosuppression, causing a lower immune response to fight off infections such as COVID-19 [145]. Chronic use has been shown to particularly affect immune dysregulation in the lungs which can be a major concern in those with COVID-19 [146]. Methamphetamine users are at increased risk due to the likelihood that they already suffer from existing diseases, weakened immunity, poor decision making, and impaired judgment, all of which can increase their susceptibility to infection [140]. Methamphetamine users have a weakened immune system which makes them susceptible to opportunistic infections [140].

The pathogenesis of methamphetamine induced lung injury is not well understood but chronic use has been linked to lung damage [147]. Inhalation of methamphetamine may be associated with an increase in free radical formation [147]. Free radical formation in the lungs as previously discussed can cause irreversible damage from chronic methamphetamine use. Damage from methamphetamine has been known to cause lung damage leading to pulmonary edema, pneumonia, and pulmonary hypertension [147]. With a majority of methamphetamine damage affecting the lungs it is critical to associate methamphetamine use with worsening outcomes in active COVID patients.

Cognitive impairments from methamphetamine use results in neurological deficits such as memory loss and changes in decision making and information processing [143]. Methamphetamine use is also associated with an increased risk of seizures, coma, agitation, anxiety, depression, paranoia, and psychosis [143]. COVID is also associated with neurological symptoms during active infection and long after eradication of the virus from the body in some patients. The term “covid fog/brain” has been widely used since the beginnings of the pandemic to describe consequences of the virus for descriptions of cognitive impairment [148]. People described this as a slowing down in their speed of information processing and decreased short-term memory. Mood and sleep disorders are also common of “long covid” with symptoms lasting over 4 weeks after illness [148]. With COVID-19, disease symptoms can continue for extensive periods after the infection’s resolution. As many as 87 % of people reported having at least one persistent symptom after 4 weeks of infection resolution [148].

With chronic use of methamphetamine, brain damage predisposes these patients to both neurologic and pulmonary COVID complications. Further research is required to see if neurological deficits related to methamphetamine use could put people at risk of developing long term complications of COVID such as the cognitive impairments mentioned above. Due to methamphetamine and COVID targeting similar organs, the brain and lungs, a correlation could be drawn that each will negatively affect the other. Brain and lung damage in methamphetamine users could impact COVID infection and its consequences. Just as in people with chronic lung conditions such as asthma or chronic obstructive pulmonary disease, people who use methamphetamine may have negative impacts on disease progression with COVID-19 due to previous damage from methamphetamine.

## Effect of cocaine use on SARS-CoV-2

In 2021, a national survey was used to conduct the usage of cocaine among people 12 years or older in the United States. In the last 12 months, there were around 4.8 million people that reported the use of cocaine [149]. In the United States, cocaine is a schedule 2 drug, with a high potential for abuse. It is one of the most powerful addictive stimulants used globally. During the COVID-19 pandemic, there has been an increased surge of people experiencing depression or anxiety, leading to cocaine use [136].

### Molecular effects

Cocaine is an addictive substance, that with excess consumption causes major complications. The exposure side effects of cocaine use are seen in many areas in the body, including the brain, cardiovascular, lungs, and immune system [149]. The abuse of cocaine activates a specific innate immune response that leads to major cellular toxicities in the brain, heart, and other respective organs. The activation of these components alters homeostasis in the CNS system and increases the production of the of pro-inflammatory cytokine IL-6 and then decreases the expression of the anti-inflammatory cytokine IL-10 [150]. Several studies have shown that cocaine can promote the oxidative stress in the organs mentioned above, hence increasing the reactive oxygen species production [150]. It is important to understand the role of these inflammatory cytokines due to the concern for patients with cocaine abuse that also have a COVID-19 infection. Cocaine affects the immune system by stimulating the hypothalamic–pituitary–adrenal axis which affects the antibody formation, lymphocyte proliferation, macrophage and NK activation; causing high incidence of viral infection [76]. This is a concern in SARS-CoV-2 due to a clinical study showing that there was a significant rise in the levels of different cytokines. In COVID-19, the cytokine IL-6 showed high levels of interest due to secreting the protein in the lung epithelial cell [151]. Due to similar synergistic effect with COVID-19, there is a major concern with the increased risk of infections, pneumonia, pulmonary embolism and asthma exacerbation [152].

As mentioned, COVID-19 and the use of cocaine concomitantly leads to an increase of inflammatory markers, leading to a higher risk of complication for the patient. The next section will bring into account the need to discuss the organs that are affected by the combination of both stimulant and virus.

### Systemic/organ effects

Cocaine can be administered in many different ways, including intranasally, inhalation or intravenously. Patients with a cocaine use disorder can experience many side effects by the usage of cocaine only. When combined with the COVID-19 infection, these side effects can lead to major complications. As mentioned, patients with COVID-19 who have a cocaine use disorder will have a greater chance of having COVID-19 complications, but this does not correlate with decreased life expectancy [136]. A study showed that patients with COVID-19 and cocaine use disorder had worse outcomes in death and hospitalization than patients without cocaine use disorder [153]. Cocaine use is associated with effects on organs throughout the body, including the cardiovascular, CNS, and respiratory systems.

Cocaine stimulates the sympathetic nervous system leading to the inhibition of catecholamines re-uptake, affecting the cardiovascular system [154]. Additionally, cocaine increases myocardial oxygen demand by increasing the patient’s heart rate and blood pressure. A retrospective cohort study showed that patients with cocaine use disorder diagnosed with COVID-19 had a further risk of new diagnosis of endocarditis [155]. The synergistic effect of COVID-19 and the consumption of cocaine has a prothrombic and hypercoagulable effect increasing the risk of myocardial infraction [156].

Cocaine can also affect the respiratory system therefore exacerbating the synergistic effect of COVID-19 in the lungs. After developing cocaine toxicity, some pulmonary complications are seen, with patients experiencing shortness of breath, wheezing, and chest pain [157]. In patients with active cases of COVID-19, these symptoms can be exacerbated, increasing the risk of pulmonary complications in these patients [158].

People with a cocaine use disorder are considered more susceptible to the infection of SARS-CoV-2 which has brought into concern the neurological symptom as well. Cocaine produces its effects in the brain by stimulating the mesolimbic dopamine system causing similar symptoms in COVID-19. SARS-CoV-2 and cocaine have a synergistic effect on the blood brain barrier (BBB), increasing the permeability to toxins, therefore causing an increased risk in the central nervous system. The disruption of the BBB permeability exacerbates the symptoms encountered in both cocaine use and COVID-19 [76]. The damage of the endothelial cells in the brain is due to the entrance of both cocaine and COVID-19 through the angiotensin-converting enzyme 2 receptors. The activation of these pro-inflammatory markers in the brain will cause patients to experience neurotoxic effects [159]. This causes more concern and monitoring for patients with cocaine use disorder and COVID-19 [159].

## Cannabinoids and COVID-19

The purchase of CBD, a component of the cannabinoid family, has increased in popularity due to availability without a prescription. CBD is being used for many different reasons, including stress, anxiety, sleep and pain. Increased ease of access has led to increased consumption. There is, however, concern for the side effects of CBD due to limited data in their safety and efficacy [160].

The properties of cannabinoids depend on their interaction with the endocannabinoid system including G-proteins coupled receptors and transient receptor potential channels [161]. CBD1 and CBD2 have a role in immunity by suppressing T-cell activation, inhibiting IL-17 secretion and regulation of intestinal neutrophils [161]. CBD can reduce proinflammatory cytokines IL-2, IL-6, IL-1α and β, interferon gamma, and inducible protein-10 that have been associated with SARS-CoV-2 [160]. Cytokines previously mentioned play a central role in the inflammatory response to COVID-19.

Pre-clinical studies show that some cannabinoids may have an impact on the inflammatory response in mouse models of lung or inflammatory diseases [161]. Inhibition of key pro-inflammatory cytokines TNFα and IL-6 were found in this study along with decreased levels of cytokines IL-5, IL-4 and IL-13 by CBD via the CB1 receptor [161]. This led to a reduction in airway inflammation, a key problem with COVID-19 infections. Cannabinoids may hold the potential for avoiding cytokine release which is responsible for most of the inflammatory mediated effects of COVID-19.

One study assessed antiviral activity of CBD and Δ9-THC against SARS-CoV-2. The study showed blocking of viral translation of SARS-CoV-2 and reducing pro-inflammatory cytokines levels in the lungs by acting as agonists of the CB-2 receptor [162]. Δ9-THC acts as a partial agonist of CB1R and CB2R and reportedly induces immunological and anti-inflammatory effects via activation of CB2R [162]. Cannabinoids are supported by evidence to be an addition to current COVID-19 therapies [8].

Other studies have assessed if CBD could help with the rise in mental health conditions from the COVID-19 pandemic. Due to the pandemic an unprecedented outcome was established between the pandemic and neurological complications such as anxiety and depression. An estimate of 45 % of adults in the US reported that their mental health was affected due to the COVID-19 pandemic [15]. Due to lack of evidence or well-designed clinical trials, data does not suggest a benefit of CBD on mental health conditions such as anxiety or depression [15].

In a study on the interaction between cannabinoids and SARS-CoV-2 spike protein, an interaction was found that two cannabinoids, cannabidolic acid and cannabigerolic acid, may block infection of human cells by SARS-CoV-2 expressing the spike protein [163]. Blocking viral attachment to human cells helps decrease viral replication and viral infection.

The anti-inflammatory properties of CBD may be beneficial for preventing worsening of disease from inflammation due to COVID-19, but caution is needed regarding the risk of abuse with cannabinoids and guideline recommended alternative therapies with no risk of abuse or addiction unlike cannabinoids.

## Conclusions

Dependence on drugs of abuse is associated to increased morbidity and mortality [17, 19, 22]. These substances when abused can affect the respiratory, cardiovascular, and psychological systems. SARS-CoV-2, the causative agent of COVID-19 also affects these organs and systems, particularly the respiratory system [17, 19, 22]. The possibility and potential for these two epidemics and pandemic to cause worsened disease outcome in persons with SUD needs to be understood. We have reviewed the molecular and organic/systemic impact SARS-CoV-2 has on individuals with substance use disorders.

Chronic alcohol consumption leads to increased SARS-CoV-2 infectivity and viral susceptibility. Alcohol consumption increases ACE2 expression, the entry point of the pathogen while altering proinflammatory cytokines [34, 38]. Heavy alcohol use is also associated with respiratory system decline by altering macrophages and lymphocyte recruitment which is essential in clearing SARS-CoV-2 pathogens [30]. This results in respiratory system decline for persons with AUD.

Although some studies suggest that tobacco (nicotine) may protect the lungs against SARS-CoV-2 by restoring the NCS and anti-inflammatory pathway, more recent studies suggest otherwise [66]. Nicotine has been found to upregulate ACE2 expression in the lower respiratory tract, the entry point for SARS-CoV-2 and increase viral infectivity by altering the immune functions [56].

Similarly, studies on marijuana (cannabis) use suggest it may have some therapeutic effect in protecting the lungs by acting as an immunomodulatory agent in suppressing cytokine storm triggered by SARS-CoV-2 [83–85]. Although this is confirmed by the decreased odd ratio of contracting the pathogen than in people with opioid, tobacco and alcohol use disorders, the risk is much greater than people with no history of substance abuse [107]. Marijuana may have some therapeutic effect against SARA-CoV-2, but its side effect should not be disregarded in terms of drug-drug interaction against other COVID-19 medications [90].

The vulnerability of people with opioid use disorders put them in a comprised health situations when infected with SARS-CoV-2 [116]. Although there has not been any research conducted at the molecular or organic/systemic level to observe the direct effect, there is a suggestion that the combination of the individual health repercussion in OUD and SARS-CoV-2 infection could lead to aggravated complications in these individuals. Considering the high rates of respiratory depression and kidney impairment in individuals with opioid use disorders and COVID-19 patients, a patient with OUD affected with the disease could be detrimental [129].

Methamphetamine and cocaine have similar detrimental effects on outcomes in patients with COVID-19. Effects on immune suppression and pulmonary toxicity seen in methamphetamine and cocaine users negatively impact patients with poor health outcomes with infection of SARS-CoV-2. Immune suppression predisposes this population to an increased risk of infections and a lesser ability to fight off the infection while pulmonary alterations can lead to worsening pulmonary complications as seen with COVID-19.

Finally, cannabinoids have been shown to have anti-inflammatory properties as well as inhibiting viral attachment to human cells, which helps decrease the likelihood of infections with SARS-CoV-2. While certain cannabinoids show a benefit with inflammation due to viral infections, large concerns include lack of data on safety and efficacy of cannabinoids especially those taken without the Food and Drug Administration approval or oversight.

People with substance use disorders have comprised health conditions which makes them more susceptible to pathogen infectivity. Considering this factor, there could be some synergistic effect that poses as a potential cause for worsened disease outcome in these population. However, more research must be conducted to indicate a direct association of the effect SARS-CoV-2 has on persons with substance use disorders.
